# Pharmacokinetics of tigecycline in critically ill patients with liver failure defined by maximal liver function capacity test (LiMAx)

**DOI:** 10.1186/s13613-020-00707-2

**Published:** 2020-08-04

**Authors:** Rawan Alraish, Sebastian G. Wicha, Otto R. Frey, Anka C. Roehr, Johann Pratschke, Martin Stockmann, Tilo Wuensch, Magnus Kaffarnik

**Affiliations:** 1 Department of Surgery, Charité - Universitätsmedizin Berlin, Campus Charité Mitte/Campus Virchow-Klinikum Augustenburger Platz 1, 13353 Berlin, Germany; 2 Dept. of Clinical Pharmacy, Institute of Pharmacy, University of Hamburg, Bundesstr. 45, 20146 Hamburg, Germany; 3 Clinical Pharmacy, Klinikum Heidenheim, Schlosshaustraße 100, 89522 Heidenheim, Germany

**Keywords:** Tigecycline, Liver function test, LiMAx, Pharmacokinetics

## Abstract

**Background:**

In critically ill patients, tigecycline (TGC) remains an important therapeutic option due to its efficacy against multiresistant Gram-positive and Gram-negative bacteria. TGC is metabolized and eliminated predominantly by the liver. Critical illness-induced liver failure may have a profound impact on the pharmacokinetic of TGC. In the present study, we aimed to establish a link between the degree of liver dysfunction and TGC plasma concentration using the novel maximum liver function capacity (LiMAx) test, as a dynamic liver function test.

**Materials/methods:**

The prospective study included 33 patients from a surgical ICU with the clinical indication for antibiotic therapy with TGC. The patients received 100 mg loading dose of TGC followed by intermittent standard doses of 50 mg q12. Blood samples for TGC plasma concentration were collected at 0.3, 2, 5, 8 and 11.5 h in a steady-state condition after at least 36 h post-standard dosage. The results were analyzed by means of a high-performance liquid chromatography (HPLC) method. Within the same day, the LiMAx test was carried out and routine blood parameters were measured.

**Results:**

Peak plasma concentrations of TGC were significantly higher in patients with severe liver failure (LiMAx < 100 µg/kg/h) when compared to patients with normal liver function (LiMAx > 300 µg/kg/h). The pharmacokinetic curves revealed higher values in severe liver failure at any measured point. Moreover, LiMAx and total bilirubin were the only liver-related parameters that correlated with TGC *C*_max_.

**Conclusions:**

The present study demonstrates a high variability of TGC plasma concentrations in critically ill patients. The results show a significant correlation between the degree of liver dysfunction, measured by the LiMAx test, and TGC *C*_max_. LiMAx test may be a helpful tool beyond others for adjusting the required dosage of hepatic metabolized antibiotics in critically ill patients.

*Trial registry* DRKS—German clinical trials register; Trial registration number: DRKS00008888; Date of registration: 07-17-2015; Date of enrolment of the first participant to the trial: 12-10-2015

## Background

Patients in the intensive care unit (ICU) are prone to develop bacterial infections, followed by an indication for antibiotic therapy. Due to altered pathophysiology in critically ill patients, finding the appropriate antimicrobial dosing is challenging with the risk of a drug over- or under-dosing, which may result in poor clinical outcome [1]. Tigecycline (TGC) is a glycylcycline antimicrobial agent, approved in 2005 by the United States Food and Drug Administration (FDA) for the treatment of complicated intra-abdominal and skin-structure infections [2–4]. It shows an expanded broad-spectrum activity against important and relevant sensible and multiresistant Gram-positive and Gram-negative bacteria such as (methicillin-resistant) *Staphylococcus aureus* (MRSA), (vancomycin-resistant) *Enterococci* (VRE) and (extended-spectrum β-lactamase-producing) *Enterobacteriaceae* (ESBL) [5]. TGC is considered as a last resort option for difficult-to-treat infections. However, data regarding suboptimal TGC dosing indicate a correlation with an increased risk of death [6]. Consequently, several clinical studies are heading towards a high-dose regimen of TGC therapy as an approach to increase its efficacy [6, 7].

On the other hand, understanding of the altered renal or hepatic function can be an alternative approach in adjusting antibiotic dosing. For instance, hepatic function plays a role in the clearance of TGC, as almost 60% of TGC is eliminated primarily via biliary excretion and approximately 20% is metabolized by the liver [6, 8, 9]. Due to a lack of reliable liver function tests, it is difficult to obtain sufficient data guiding clinicians in TGC dose adjustment in critically ill patients with liver dysfunction.

The maximal liver function capacity (LiMAx) test has been recently introduced as a non-invasive diagnostic tool for determining acute liver failure in the intensive care medicine [10]. It determines the enzyme activity of the liver based on a non-invasive breath test. Concerning antibiotic dosing of non-renal eliminated drugs, one recent study demonstrated a correlation between LiMAx and the pharmacokinetics (PK) of linezolid [11].

In our study, we aimed to examine the impact of liver dysfunction on the PK of TGC in critically ill patients using the novel LiMAx test.

## Methods

### Patients and study design

This current study was approved by the ethics review board of the Charité medical faculty (EA4/022/13) in accordance with the provisions of the declaration of Helsinki. Prior to study inclusion, written informed consent was obtained from all participants or their responsible legal representatives.

Patients were recruited from the surgical ICU of the Charité University Hospital, Berlin, Germany. Inclusion criteria were a medical indication for intravenous anti-infective therapy with TGC and an age between 18 and 99 years. Exclusion criteria were an allergy against TGC or methacetin, co-medication with substances metabolized by cytochrome P450 1A2 or with substances affecting the clearance of TGC or missing informed consent. Microbiological samples were obtained from any suspected source of infection, as well as from blood (in duplicate for anaerobic and aerobic testing) prior to the first TGC dosages. The samples were sent to a central laboratory for verification of the causative pathogen, minimal inhibitory concentrations (MIC) breakpoints determination and storage. MIC values were determined by broth microdilution testing (BMT). According to the current guidelines, patients with septic shock or detection of TGC-resistant pathogens in the microbiological samples were treated with TGC only in combination with other broad-spectrum antibiotics [12].

Patients received an initial dose of 100 mg of TGC in a 30 min infusion, followed by multiple doses of 50 mg/30 min q12. In order to measure the plasma concentrations of TGC in a steady-state condition, measurement and probe sampling were carried out at least 36 h after the first dosage of TGC. On the same day, the LiMAx test was performed according to recent publications and serum samples for static liver function parameters and routine blood parameters were taken [11, 13]. Static liver function parameters included aspartate aminotransferase (AST), alanine aminotransferase (ALT), gamma-glutamyl transferase (GGT), pseudocholinesterase (PCHE), glutamate dehydrogenase (GLDH), alkaline phosphatase (ALP), total bilirubin, lactate, platelets count and international normalized ratio (INR). For pharmacokinetic analysis of TGC, 5 mL of heparinized blood samples were collected at 0.3, 2, 5, 8 and 11.5 h after the end of TGC infusion. Within 1 h after collection, blood samples were centrifuged at 4000 rpm for 5 min and plasma aliquots were stored at − 80 °C. Plasma TGC concentrations were determined using a previously described high-performance liquid chromatography method (HPLC) [14]. In addition, we conducted the model for end-stage liver disease (MELD score). The severity of illness was defined using the Acute Physiology and Chronic Health Evaluation (APACHE II) score, the Sepsis Organ Failure Assessment (SOFA) score and the Simplified Acute Physiology Score II (SAPS II).

After assessing the pharmacokinetics of TGC with the identified bacteria associated with the infection, patients were divided into three groups depending on their LiMAx value on the day of measurement according to previously published data [10]. Group A included patients with LiMAx values < 100 μg/kg/h (severe liver failure), group B patients with LiMAx values between 100 and 300 μg/kg/h (moderate liver failure) and group C patients with LiMAx values > 300 μg/kg/h (normal liver function). To correlate TGC serum levels with liver function, we compared the three different groups focussing mainly on Group A and C. To identify factors predicting TGC *C*_max_, a linear multivariate regression analysis was conducted including parameters that were found different between group A and C.

### Statistical analysis

Continuous variables were shown as median and interquartile range; meanwhile, the categorical variables were presented in frequencies. The suitable statistical test was conducted depending on the values’ distribution of each variable using Kolmogorov–Smirnov test in combination with the Shapiro–Wilk test. Comparisons in normally distributed variables were performed using the Mann–Whitney *U* test. Not normally distributed variables were tested using the independent *t* test for non-connected samples. A *p*-value of < 0.05 was considered statistically significant.

Multivariate linear regression was performed to identify parameters related to liver function predicting TGC *C*_max_ variability. These parameters were included in the final regression analysis after meeting the assumption of collinearity. This was tested using Pearson’s Chi-squared test to examine the correlation between liver function parameters with each other. The assumption was verified by observing the variance inflation factor and tolerance in the final regression analysis. Moreover, the correlation between LiMAx and TGC PK parameters were analyzed using simple linear regression. The log-trapezoidal rule was used to compute the area under the concentration–time curve (AUC) from 0 to 12 h for the mean concentration–time data in plasma. Statistical analysis was carried out using SPSS Statistics 22 (SPSS Inc., Chicago, IL, USA).

## Results

### Demographic and characteristics of patient groups

A total of 33 patients were included. These patients were divided into the three LiMAx groups as follows: (A) LiMAx < 100 μg/kg/h (*n* = 5); (B) LiMAx 100–300 μg/kg/h (*n* = 21) and (C) LiMAx > 300 μg/kg/h (*n* = 7). In all patients, the indication of TGC therapy was complicated intra-abdominal sepsis. Enterococcus faecium was the most commonly observed pathogen (33.3%) in the microbiological samples with a MIC value of 0.12 mg/L (Table 1). Forty-two percent (*n* = 14) of the causative organisms were Gram-negative with a MIC value of 0.5 mg/L (Table 1). Comparisons of the baseline characteristics between groups A and C revealed significant differences in the SOFA score (*p* = 0.019) and the BMI (*p* = 0.018) (Table 2).

**Table 1:** Pathogens associated with infections caused in the study population

	Total (*n* = 33)	Group A (*n* = 5)	Group B (*n* = 21)	Group C (*n* = 7)	MIC breakpoints (mg/L)
Microbiological isolate, n (%)					
*Enterococcus avium*	1 (3)	0	1 (4.8)	0	0.12
*Enterococcus faecalis*	3 (9.1)	2 (40)	1 (4.8)	0	0.12
*Enterococcus faecium*	11 (33.3)	4 (80)	7 (33.3)	0	0.12
*Escherichia coli*	4 (12.1)	2 (40)	2 (9.5)	0	0.5
*MRSA*	1 (3)	0	0	1 (14.3)	0.25
*Staphylococcus epidermidis*	2 (6.1)	0	3 (14.3)	1 (14.3)	0.12
*VRE*	9 (27.3)	2 (40)	3 (14.3)	4 (57.1)	0.12
*ESBL*	10 (30.3)	3 (60)	4 (19)	3 (42.9)	0.5

MRSA: methicillin-resistant *Staphylococcus aureus*; VRE: vancomycin-resistant Enterococci; ESBL: extended betalactamase producing Gram-negative bacteria; MIC: minimum inhibitory concentration

**Table 2:** Baseline characteristics

	Total	Group A	Group B	Group C	*p*^a,b,c^
Patients (*n*)	33	5	21	7	
Age (year)	63 (54–73)	59 (54–77)	65 (55–73)	59 (54–68)	0.456^a^
Gender (m/f)	20/13	3/2	14/7	¾	0.812^c^
BMI (kg/m^2^)	26.4 (22.6–31.2)	22.5 (18.3–22.7)	27.7 (24.4–31.1)	31.3 (24.5–46.4)	0.018^b^
WBC (/nL)	17 (13–22)	26 (25–34)	14 (10–21)	15 (11–35)	0.190^b^
CRP (mmol/L)	124.2 (78.3–217.3)	88.8 (61.1–197.8)	135 (106.9–176.8)	114.3 (42.2–210.8)	0.899^a^
INR	1.38 (1.24–1.54)	1.28 (1.24–1.54)	1.39 (1.28–1.56)	1.34 (1.19–1.98)	0.584^a^
Lactate (mg/dL)	15 (12–27)	30 (21–55)	15 (11–21)	15 (13–26)	0.114^b^
Platelets count (/nL)	182 (104–374)	136 (68–287)	171 (88–310)	335 (187–444)	0.127^a^
Albumin (g/L)	22.6 (19.6–25.03)	22.9 (16.8–29.2)	22.7 (18–25.4)	24 (16.8–25.2)	0.766^a^
Creatinine clearance (mL/min)	32 (20–64)	29 (0–75)	32 (21–57)	32 (25–89)	0.606^a^
SOFA score	9 (5–11)	12 (11–16)	9 (5–11)	8 (4–10)	0.017^a^
SAPSII score	55 (41–72)	57 (51–65)	59 (39–71)	55 (35–68)	0.547^a^
APACHE II score	23 (18–30)	27 (21–34)	25 (15–28)	28 (19–30)	0.698^a^
Duration of therapy (days)	8 (6–12)	7 (4–9)	9 (7–14)	6 (5–12)	0.857^b^

Data are presented as median and interquartile range (25th to 75th percentile) or frequencies

BMI: body mass index; WBC: white blood cell count; CRP: C-reactive protein; INR: international normalized ratio; SOFA: Sequential Organ Failure Assessment; SAPS: Simplified Acute Physiology Score; APACHE II: Acute Physiology and Chronic Health Evaluation; *p* value: comparison between group A and D: ^a^Independent *t* test, ^b^Mann–Whitney *U* test, ^c^Fisher’s exact test

### Pharmacokinetics of TGC

**Fig. 1:**
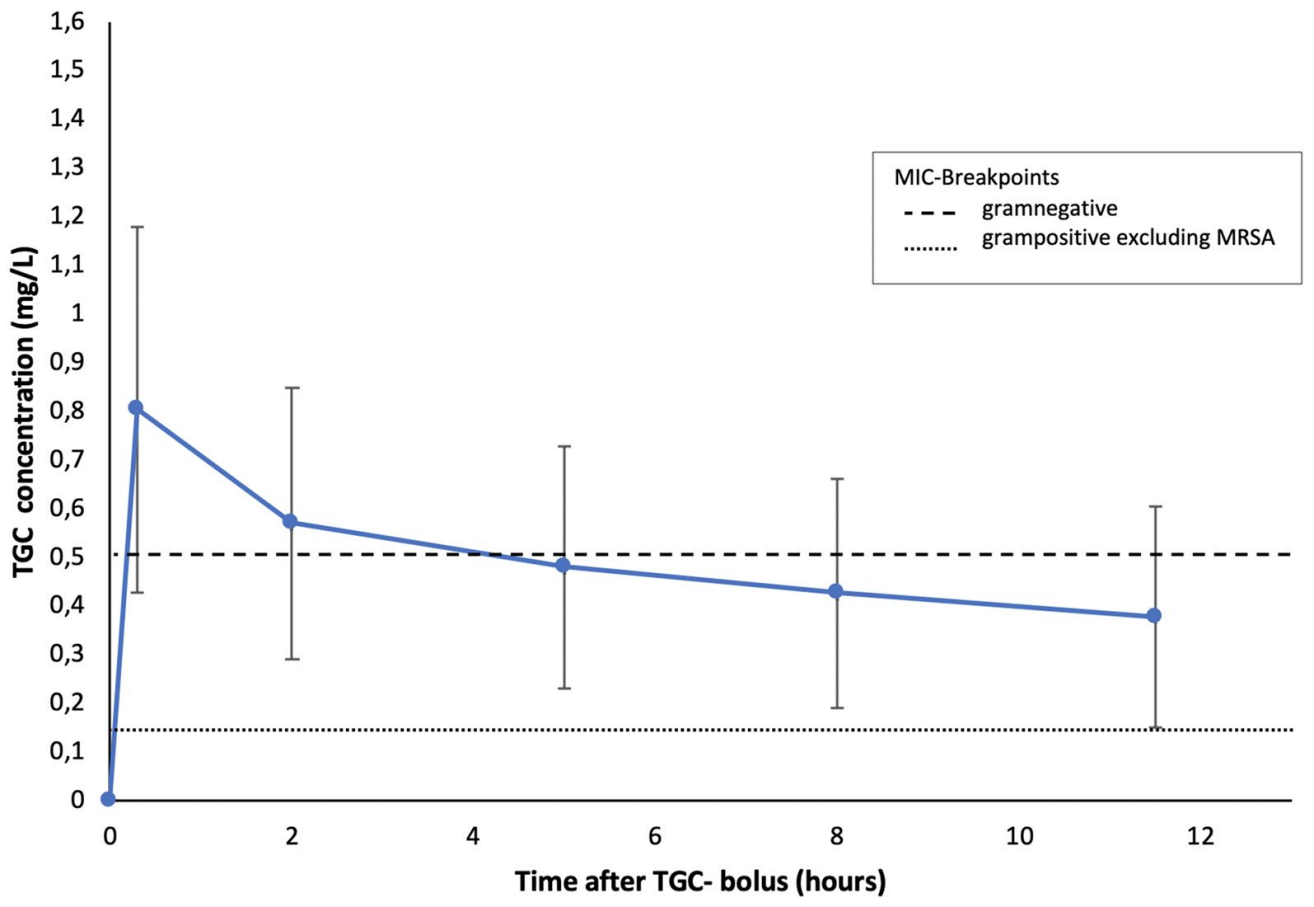
Mean TGC plasma concentrations depending in the study population. Data are presented as mean ± standard deviation

The mean TGC concentrations in plasma-versus-time profiles in the whole population are shown in Fig. 1. Mean plasma concentrations of TGC showed peak levels (*C*_max_ = 0.805 mg/L) 0.3 h after TGC bolus administration, followed by a decrease until reaching a trough level (*C*_min_ = 0.377 mg/L) 0.5 h before the next TGC bolus. A statistical summary of pharmacokinetic and pharmacodynamic exposure variables of TGC is presented in Table 3. For the Gram-positive bacteria, 99.6% of TGC dose interval was above MIC < 0.12 mg/L without MRSA, where 99.1% of the TGC was above MIC < 0.25 mg/L with MRSA. For the Gram-negative bacteria 35% of TGC dosing was above the corresponding MIC values.

**Table 3:** ﻿Summary of pharmacokinetic and pharmacodynamic parameters of TGC

Measure	*C*_max_ (mg/L)	AUC (mg h/L)		MIC (mg/L)	AUC/MIC
	0.805	5596	Gram positive without *MRSA*	0.12	46.63
			Gram negative	0.5	11.192

AUC: area under the concentration–time curve over 12 h; MIC: minimum inhibitory concentration

**Fig. 2:**
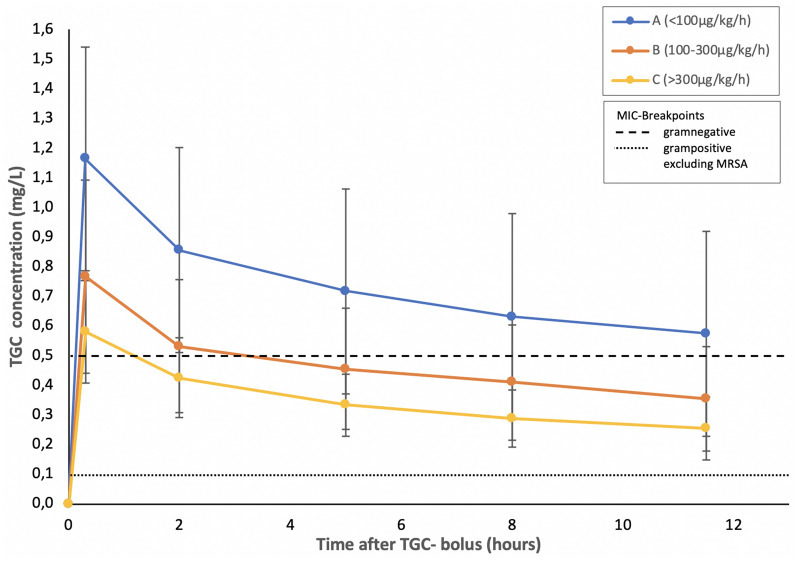
Mean TGC plasma concentrations depending on the degree of liver failure. Data are presented as mean ± standard deviation

TGC mean plasma concentration curves between groups A–C are shown in Fig. 2. The total extension of *C*_max_ ranged from 0.441 to 1.774 mg/L. The highest mean values of *C*_max_ were observed in group A and lowest in group C (1.135 mg/L vs. 0.581 mg/L, *p* = 0.004). Group A presented TGC PK curves with the highest and group C with the lowest mean values. Group B showed TGC mean values in between groups A and C. Mean values of groups A and C were significantly different at all time-dependent measuring points (0.3 h: *p* = 0.004; 2 h: *p* = 0.02; 5 h: *p* = 0.02; 8 h: *p* = 0.004; 11.5 h: *p* = 0.011). In view of the pharmacodynamics of TGC, the AUC/MIC in group A patients was higher compared to patients in group C against both Gram-negative and Gram-positive bacteria (68.383 vs. 25.827 for Gram-positive bacteria without MRSA and 16.412 vs. 7.748 for Gram-negative bacteria, *p* < 0.05).

### Hepatic dysfunction

The LiMAx test showed significantly lower values in group A compared to group C (71 μg/kg/h vs. 438 μg/kg/h, *p* < 0.001). Static liver function tests demonstrated significantly higher parameters for lactate (27 mmol/L vs. 12 mmol/L, *p* = 0.001) and INR (2.1 vs. 1.3, *p* = 0.017) in group A. Platelet counts (60/nL vs. 312/nL, *p* = 0.017) and ALP (105 IU/L vs. 271 IU/L, *p* = 0.017) revealed lower values in group A. Other parameters such as MELD score, total bilirubin, AST, ALT and GGT showed no significant difference within the groups (Table 4).

**Table 4:** Comparison of static and dynamic liver parameters

	Group A	Group C	*p*^a,b^
LiMAx (μg/kg/h)	71 (37–88)	438 (330–456)	< 0.001^b^
Lactate (mmol/L)	27 (24–30)	12 (9–14)	0.001^a^
INR	2.1 (1.5–2.7)	1.3 (1.3–1.4)	0.017^a^
Total bilirubin (mmol/L)	2.8 (1.5–5.6)	1.6 (0.6–2.2)	0.209^a^
Platelet counts (/nL)	60 (46–83)	312 (177–462)	0.017^a^
AST (IU/L)	28 (24–56)	59 (43–74)	0.097^a^
ALT (IU/L)	13 (12–39)	52 (33–94)	0.053^a^
GGT (IU/L)	67 (59–76)	202 (84–485)	0.051^a^
ALP (IU/L)	105 (83–152)	271 (143–381)	0.017^a^
MELD score	18 (16–36)	14 (11–23)	0.201^b^

Data are presented as median and interquartile range (25th to 75th percentile)

LiMAx: maximum liver function capacity; INR: international normalized ratio; AST: aspartate aminotransferase; ALT: alanine aminotransferase; GGT: gamma-glutamyl transferase; ALP: alkaline phosphatase; MELD: model for end-stage liver disease

^a^Independent *t* test

^b^Mann–Whitney *U* test

### Multivariate analysis

LiMAx and total bilirubin were the only parameters predicting TGC *C*_max_. LiMAx was negatively correlated with TGC *C*_max_, indicating higher TGC plasma levels when LiMAx values were low. Total bilirubin was positively correlated with TGC *C*_max_. Other variables (lactate, INR and MELD score) failed in correlating with TGC *C*_max_. This multiple regression model accounts for about 40% of TGC *C*_max_ variance with *F* (3.30) = 8.539 (*p* < 0.001) (Table 5).

**Table 5:** Multiple linear regression model: co-factors predicting TGC *C*_max_

	Beta	*p*-value
Constant		< 0.001
Total bilirubin (mg/dL)	0.495	0.001
LiMAx value (µk/kg/h)	− 0.293	0.044
Lactate	–	0.336
MELD score	–	0.211
INR	–	0.558

LiMAx value (maximum liver function capacity), total bilirubin, Lactate, MELD score (model for end-stage liver disease) and INR (international normalized ratio)

**Fig. 3:**
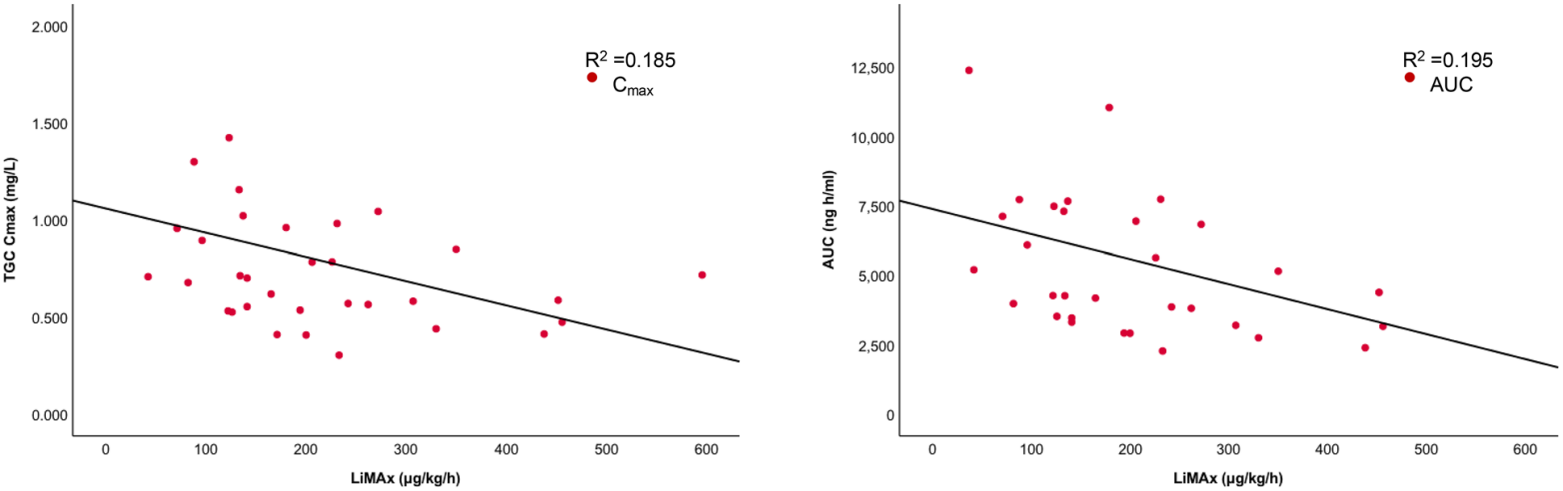
Relationship between LiMAx and TGC PK, AUC (**a**) and *C*_max_ (**b**). *C*_max_: TGC maximum plasma concentration; AUC: area under the curve

A demonstrated scatter plot revealed a strong negative linear correlation between LiMAx and TGC PK parameters. Simple linear regression showed a significant correlation between LiMAx and TGC *C*_max_ (*p* < 0.001) and between LiMAx and TGC AUC_0–12_. The *R*^2^ value for both was almost similar reaching 0.195, indicating that nearly 20% of the variation in TGC *C*_max_ and TGC AUC_0–12_ may be explained by quantifying liver function using only the LiMAx test. The scatterplot of standardized predicted values versus standardized residuals indicated that the data met the assumptions of homogeneity of variance and linearity. The residuals were approximately distributed normally (Fig. 3).

## Discussion

The present study investigated the effect of liver failure on TGC PK for the first time. Based on recently conducted trials, we established a novel strategy using the LiMAx test for quantifying hepatic dysfunction [10, 15, 16]. Our findings showed a significant increase in TGC PK curves in patients with strong hepatic dysfunction (group A), compared to patients with normal liver function (group C). The results of the multivariate analysis confirmed this effect. Regarding liver function, only LiMAx and total bilirubin revealed a significant impact on TGC *C*_max_.

TGC *C*_max_ in our patient groups ranged from 0.441 to 1.774 mg/L. They were within the range to those determined in previous clinical trials with healthy volunteers [3, 17]. However, extrapolating data of healthy subjects into critically ill patients is a challenge due to altered pathophysiology in this specific patient population. Particularly, the role of liver dysfunction is not sufficiently described, so far [1]. Moreover, limited data are published, illuminating the pharmacokinetic of TGC in critically ill patients.

The LiMAx test has previously shown promising results in quantifying hepatic function in sepsis. The investigators concluded that LiMAx was a reliable diagnostic tool for identifying liver failure in critically ill patients [10]. In another study, LiMAx explained a reasonable part of the linezolid PK variability of critically ill patients regarding the degree of liver failure. The authors demonstrated a strong association between LiMAx and non-renal clearance of linezolid by reducing the interindividual variability from 46.6 to 33.6%. LiMAx was superior to other markers of organ failure such as creatinine clearance (CLCr), thrombocyte count, total bilirubin and GGT even though an opportunistic probe sampling with linezolid trough levels was chosen in this work [11]. The results of the present study with a more sophisticated strategy reflecting the PK of TGC demonstrate that LiMAx may provide an adequate diagnostic tool for predicting high TGC plasma levels in patients with hepatic dysfunction. Particularly, the LiMAx results < 100 µg/kg/h should lead to increased attention from the physicians. In vitro, data of TGC revealed a protein binding of about 50–70% [18]. A low protein-binding of TGC yields a high volume distribution in the different body compartments and may lead to an imprecise interpretation of TGC plasma levels. Hence, a dosage algorithm based on the therapeutic drug monitoring of the TGC plasma level may be challenging to establish. Studies investigating TGC levels in other human body fluids such as bile or ascites are further required.

LiMax also provided some superior insights into the dosage regimes used across the norm. Since December 2018, EUCAST recommends a 200 mg loading dose of TGC followed by 100 mg steady-state dosage in treating critically ill patients infected with pathogens resistant to all other classes of antimicrobials [19]. In our clinical study, a standard dosage, 100 mg loading dose of TGC followed by 50 mg steady-state dosage has been used. We observed, in Gram-positive bacteria, that critically ill patients in group A (68.383) had a significantly higher AUC above the MIC values than patients in group C (25.827). Similarly, in Gram-negative bacteria, group A (16.412) had a higher AUC above the MIC values than patients in group C (7.748). In such circumstances, EUCAST recommended dosage could lead to an increase in the risk of developing resistance towards TGC.

Another dynamic liver function test is the ICG-PDR, which is more widely used in clinical settings than the LiMAx test. Several authors investigated ICG-PDR in different conditions. The majority of authors came to the conclusion that ICG-PDR may not accurately measure liver dysfunction in sepsis due to complex ICG kinetics in liver disease and temporary redistribution into extrahepatic-extravascular tissues [20]. Moreover, ICG elimination seems to be severely influenced by the splanchnic perfusion and is inhibited by hyperbilirubinemia, other anionic substances and acute cholestasis without evidence of changes in hemodynamic or morphology of hepatocytes [21–24]. In addition, a recent study investigating liver function in sepsis showed a superiority of the LiMAx against the ICG-PDR in terms of quantifying liver dysfunction in critically ill patients [10]. Such findings lead to the conclusion not to use the ICG-PDR in the recent study.

Static liver function parameters, such as AST, ALT, and GGT, failed to predict the degree of liver dysfunction accurately [25]. The results in the present study confirmed these findings. Since the values of lactate, INR, platelet count and ALP differed significantly between groups A and C, these parameters failed to predict the variability of TGC *C*_max_ in the multivariate analysis. These findings are consistent with several studies, which identified only total bilirubin as a significant covariate to describe liver function [26–28]. Other authors concluded that INR is a reliable diagnostic tool to define liver failure [29]. INR can be influenced by disseminated intravascular coagulation or secondary hemorrhages, which are common complications in critically ill patients. A previous study in patients after major abdominal surgery showed similar postoperative progress of INR readouts compared with LiMAx values, while total bilirubin failed in predicting liver failure [15]. These heterogeneous results of different studies investigating the accuracy of static liver function parameters in defining liver function, point out weak reliability of these parameters in this specific issue.

Besides the described dynamic and static liver function tests, other tools are introduced to define liver failure. Korth-Bradley et al. described a significant increase in TGC *C*_max_ and AUC in patients with advanced liver cirrhosis. The diagnostic tool used to define the degree of liver cirrhosis was the Child–Pugh score [30]. However, the Child–Pugh score in critically ill patients may not be suitable to describe liver dysfunction reliably [31]. In a systematic review, Cholongitas et al. mentioned that the Child–Pugh score in ICU patients can respond rapidly under treatment, resulting in an increase from class C to A. This apparent accelerated improvement might result in adjusting antibiotic dosage unnecessarily with the possible consequence of overdosing and side effects [32]. Hence, guiding antibiotic therapy according to the Child–Pugh score in critically ill patients appears to be imprecise [33]. Based on these data, we decided not to use the Child–Pugh score in the present study.

Another tool targeting liver failure is the MELD score. Initially, the MELD score was evaluated for patients undergoing a transjugular intrahepatic portosystemic shunt [34]. The current version includes three objective variables (total bilirubin, INR and creatinine) and is simple to assess. The MELD score is predominantly used to prioritize the receipt of a liver transplant. Recent studies evaluated the MELD score in different clinical situations with positive results in patients with heart failure [35]. In the present study, the MELD score revealed no differences between groups A and C, and the multivariate analysis showed no significant impact of the MELD score on TGC *C*_max_. Hence, the MELD score appears not to be a reliable diagnostic tool to quantify liver dysfunction in critically ill patients.

In the present study, BMI was one parameter showing promising differences between the study groups at baseline. Patients of group A (high *C*_max_) revealed a significantly lower mean BMI (26.4 kg/m^2^) compared to patients of group C (low *C*_max_, BMI 31.3 kg/m^2^). The low protein binding and high distribution of TGC may be one possible explanation of this effect. However, in the multivariate analysis, BMI failed to qualify as a predictor of TGC variability. These findings are in concert with the results of other authors. Xie et al. found in their study, that BMI was an important parameter influencing the total CL of TGC. The authors pointed out that in their model building process, the simulations were beyond the BMI of the patients included and should be considered cautiously [36]. On the other hand, Pai et al. characterized the concentration profiles of TGC in the serum and urine of obese and normal-weight healthy adults and found no differences [37]. In accordance with these data, BMI may provide as one parameter influencing TGC PK, but the exclusive impact on TGC distribution appears weak.

## Conclusion

The results of this study indicate that TGC plasma levels show wide variability in critically ill surgical patients. Since TGC is eliminated predominantly non-renal, liver dysfunction may be a critical reason for TGC variability. The correlation with the results of the LiMAx test confirms these findings and LiMAx may provide an adequate tool to determine the impact of liver dysfunction on the PK of TGC. LiMAx in combination with other diagnostic tools determining organ failure in critically ill people may enhance the individual dosage of anti-infective drugs, resulting in a better outcome for the patients.

## Data Availability

The datasets analyzed during the current study are available from the corresponding author on reasonable request.
